# How does long‐term care impact the psychological wellbeing of older adults in different care policy contexts in the Netherlands? A comparison of 1998, 2008 and 2018

**DOI:** 10.1111/hsc.13719

**Published:** 2022-01-17

**Authors:** Jens Abbing, Bianca Suanet, Marjolein Broese van Groenou

**Affiliations:** ^1^ Department of Sociology Vrije Universiteit Amsterdam Amsterdam The Netherlands

**Keywords:** depressive symptoms, informal care, long‐term care, perceived care sufficiency, psychological wellbeing, unmet care needs

## Abstract

Receipt of long‐term care (LTC) is generally associated with worse psychological wellbeing for community‐dwelling older adults. In addition to objective features of care use (e.g. formal vs. informal care), the subjective evaluation of care provision in terms of perceived sufficiency might be particularly predictive of one's wellbeing but is seldomly considered in the literature. Substantial changes in the availability of long‐term care in past decades raise the question to what extent these effects, if present, are consistent over historic time. The present study, therefore, aims at better understanding the associations between types of LTC use and perceived care sufficiency on psychological wellbeing in a changing LTC context in the Netherlands. Data from the Longitudinal Aging Study Amsterdam (LASA) were used from three points in time: 1998 (*N* = 582), 2008 (*N* = 459) and 2018 (*N* = 415). At each wave, participants were between 75 and 85 years of age and living independently. The results show that after adjusting for age, gender, education and health, using formal LTC had a negative effect on depressive symptoms only in 2018, but that this effect was not significantly worse compared to previous cohorts. Perceived care sufficiency was consistently negatively associated with depressive symptoms in all three points in time. This suggests that despite a less generous Dutch LTC system, psychological wellbeing among LTC users remains stable. Perceiving care provision as sufficient, however, can help older adults maintain psychological wellbeing and should be considered by researchers and policymakers that aim to improve care recipients’ wellbeing.

**What is known about this topic?**

- Formal care use has been associated with worse psychological wellbeing.
- Unmet care needs have been associated with worse psychological wellbeing.
- Recent reforms in LTC systems resulted in less use of informal and formal care.

**What this paper adds?**

- Formal care use has a comparable negative effect on psychological wellbeing in 1998, 2008 and 2018.
- Perceived care sufficiency is highly and positively associated with psychological wellbeing.
- Perceiving care as sufficient has become less important for psychological wellbeing.

## 1 INTRODUCTION

When age‐related health impairments emerge and managing daily lives becomes difficult, maintaining psychological wellbeing can become challenging for older adults. As a response, they might choose to acquire assistance in the form of long‐term care (LTC, assistance in personal or household activities due to the care recipient's health‐related inability to perform these tasks). However, this can lead to an undesirable dependency on others (Fine & Glendinning, 2005; Stewart & McVittie, 2011), and as result, LTC recipients might struggle to maintain their psychological wellbeing (PWB), i.e. to retain a positive affective state of mind. This is indicated by the prevalence of depressive symptoms, which indicate affective problems (e.g. worrying, sleeplessness) and are often prevalent in LTC recipients (Pot et al., 2005). However, having unmet care needs (Pepin et al., 2017) and receiving insufficient care (Broese van Groenou, 2020) can be similarly detrimental to one's PWB, which highlights the importance of the subjective evaluation of LTC.

Both the use of various types of care and its perceived sufficiency may differ with the specifics of the LTC context, which includes the allocation, accessibility, and adequacy of publicly provided care. As a result of the rising demand for LTC, for example, budget‐cuts were introduced in many countries that limit the use of such care (Colombo et al., 2011; Spasova et al., 2018). This may have led to more older adults perceiving their care provision as insufficient and, consequently, experiencing worse PWB. At the same time, policy measures are also aimed at and providing care that is more efficient and closer to recipients’ needs (Janssen, Jongen, & Schroder‐Back, 2016). An open question is to what extent the importance of care use and its perceived sufficiency for wellbeing has remained stable in the light of these changes. Sufficient care provision likely remains equally beneficial but acquiring adequate LTC that allows maintenance of PWB might have become more challenging. The aim of the present study, therefore, is to investigate the robustness of the relationships between LTC use, its perceived sufficiency and psychological wellbeing over historic time. In order to capture these trends, home‐dwelling older adults’ (aged 75 to 85) types of LTC use, perceived care sufficiency and PWB are compared across three points in historical time (1998, 2008 and 2018), in which Dutch LTC policies greatly differed from one another. The knowledge gained from this present study allows conclusions about the universality of this relationship by taking different LTC contexts into account. This knowledge is also valuable for policymakers aiming at identifying effective measures to promote the wellbeing of older care recipients.

### 1.1 The effect of types of LTC use on psychological wellbeing

When individuals with health impairments receive LTC, they become dependent on others. As having a sense of control over one's life is critical for maintaining wellbeing in later life (De Quadros‐Wander et al., 2014), losing this sense of control can feel undesirable (Fine & Glendinning, 2005; Stewart & McVittie, 2011) and lead to emotional distress (Newsom & Schulz, 1998), thus compromising PWB. In addition, when care recipients feel they are placing a burden on others (McPherson et al., 2011), this can also lead to depressive symptoms, further compromising their PWB (Cukrowicz et al., 2011). So, the use of LTC is generally associated with worse PWB (Pot et al., 2005).

One of the crucial factors in the impact of LTC use on PWB is the source that provides LTC. As informal, formal and privately paid care impose different restrictions on care recipients, their perceived control likely varies as a result, inducing differences in PWB between care recipients. When receiving formal care, the recipient becomes dependent on governmental regulations. Consequently, his or her options regarding the amount and frequency of LTC as well as the person that provides LTC are limited as these choices are determined by home‐care providers. Even if changes in the care arrangement are possible, formal care recipients often struggle with the necessary administrative procedures (Zuurmond & de Boer, 2020) and therefore often choose to retain the existing arrangement (Marangos et al., 2018). Consequently, Pepin et al. (2017) found an elevated prevalence of depressive symptoms in recipients of formal care and mention losses of independence as likely causes.

In contrast, if care recipients buy LTC services with their own financial means (privately paid care) instead of receiving publicly funded formal care, they are able to decide when and how care is provided, often in direct negotiation with the care provider. In case of dissatisfaction, they can turn to another care provider without excessive administrative procedures. This provides LTC recipients with more control over the care arrangement, which might be one of the reasons for the higher psychological wellbeing among recipients of privately paid care (Broese van Groenou, 2020).

Receiving only informal care differs from formal and privately paid care arrangements: The care providers, in most cases spouses or children, already have an existing informal and often affective relationship with the recipient. They can discuss the nature of care provision personally and independently of the regulations of professional care, leaving the care recipient more in control. However, recipients of informal care might struggle with placing a high burden on caregivers, in particular on filial caregivers who often have to combine care responsibilities with occupational and family responsibilities. Although spousal care comes more naturally and expected, the care recipient may also be aware that their spouses provide many hours of care and feel overburdened (Juntunen et al., 2018; Pinquart & Sörsensen, 2011). Due to concerns about overburdening their loved ones, care recipients often refuse to ask them for assistance (Bredewold et al., 2020) or experience poor PWB when using informal care.

In summary, we expect that receiving formal care is most detrimental to one's PWB, followed by non‐spousal informal care and then by spousal care, while we expect care received from privately paid caregivers to be the least detrimental (Hypothesis 1).

### 1.2 The effect of perceived sufficiency on psychological wellbeing

If care provision is perceived as insufficient, recipients likely struggle with activities of daily living (Na & Streim, 2017). Recipients value the support that enables them to, e.g., eat or shower as frequently as they desire (Komisar et al., 2005), and that provides them with a feeling of safety and attentiveness (De São José et al., 2016). Daily hassles that might arise if these needs are not met can have a detrimental impact on wellbeing (Landreville & Vézina, 1992). The link between perceived sufficiency and caregiving is also acknowledged by recipients, as 94% of institutionalised LTC recipients in a recent Dutch evaluation have indicated that the care they receive contributes to their wellbeing (Zuurmond & de Boer, 2020). We, therefore, expect that perceived care sufficiency has a positive impact on PWB (hypothesis 2).

### 1.3 Changes over time

The relationship between LTC use, care sufficiency and PWB might have changed as a result of the substantial policy changes in the Netherlands from a generous LTC provision in the late nineties, to de‐institutionalisation in the mid‐2000s, and the substantial reorganisation of the LTC system since 2015.

In the late nineties, long‐term care was covered by the Dutch Exceptional Medical Expenses Act (AWBZ) compulsory insurance against the risk of long‐term care. LTC provision was universal and generous (Kraus et al., 2010) the number of beds in nursing homes was among the highest within EU countries (OECD, 2021a), but LTC at home was also provided generously. Thus, access to LTC was self‐evident for those that needed assistance.

However, Dutch LTC expenditure was also far above the EU average (Eurostat, 2021). Therefore, during the following decade, de‐institutionalisation policies were implemented to contain the increasing costs of LTC (De Meijer et al., 2015). Older adults were expected to stay at home as long as possible, which led to a decrease in the number of LTC beds per 1000 over 65‐year‐olds from 81.4 in 1998 to 70.1 in 2008 (OECD, 2021a) and an expansion of home‐care provision (da Roit, 2012). During the early and mid‐2000s, policies were implemented that aimed at incentivising time‐efficient LTC provision by care providers, but also at giving clients more choice in arranging LTC according to their preferences (Mot et al., 2010). More focus was also placed on family‐ and communal care responsibilities, evident by formally requiring co‐residing family members to provide informal care since 2003 and shifting LTC responsibilities from the national level to municipalities with the 2007 Social Support Act (Da Roit, 2012).

These policy trends were reinforced by the fundamental reorganisation of the LTC system in 2015. The AWBZ was abolished and in part replaced by the Long‐Term Care Act, which limited the eligibility for residential care to the most severe cases needing 24/7 care. This increased the demand for care at home and the ability to arrange that locally. Also, the Social Support Act was extended and more LTC services were now provided by municipalities (Janssen et al., 2016). Despite the aim to arrange care more at home for the ageing population, the number of over 65‐year‐olds that received publicly provided care at home decreased drastically from 383,207 in 2014 to 261,905 in 2015 (OECD, 2021b).

Thus, the situation for those in need of LTC has changed substantially over the past three decades: LTC resources are scarcer, which not only reduces older adults’ access to LTC but also impacts the experience of those that receive LTC. During the 2000s, policy changes might have successfully attenuated some of the potential negative consequences for older adults’ PWB. Policies that improve client choice, such as the personal budget, were positively received (Knijn & Verhagen, 2007), and the expansion of home‐care is in line with older adults’ preference to remain living independently. However, the reduction of formal home‐care after 2015 might be particularly detrimental to older adults’ PWB, as they may struggle to receive sufficient and adequate assistance. Concerns have indeed been raised that turning to informal care use is a result of having no other options rather than a result of changing norms towards social responsibility (Oudijk et al., 2011). Furthermore, substituting formal with informal care might be inadequate to meet recipient's care demands (Bonsang, 2009).

Policy changes aiming at improving efficiency and client choice might attenuate some of these negative effects, but critical evaluations of the reform also called into question whether these policy goals are met (Alders & Schut, 2019; Van Ginneken & Kroneman, 2015; Maarse & Jeurissen, 2016). The potential benefits of these policy changes might also become exhausted: While older adults might appreciate incremental changes towards more client choice, this might be perceived as burdensome when LTC is difficult to acquire but recipients are still expected to take responsibility for arranging the care they need.

We, therefore, expect that the association between LTC use and PWB became more negative over time for those receiving non‐spousal informal, privately paid, and formal care, but less so for those that receive spousal care (hypothesis 3a). For formal care, this might be attributed primarily to increasing quality problems, while non‐spousal care and privately paid care use might be increasingly forced due to the lack of formal care. In contrast, perceiving care as insufficient is likely similarly problematic across cohorts as it always indicates that important LTC needs are not met. We, therefore, expect that the positive effect of perceiving LTC provision as sufficient remains robust over time (hypothesis 3b).

### 1.4 Other predictors of wellbeing

There are several other factors that are associated with both LTC use and PWB and can be more or less prevalent in a 75–85‐year‐old cohort. First, LTC use is primarily a result of health impairments (Andersen & Newman, 2005), while better physical health (Steptoe et al., 2015) and cognitive functioning (Llewellyn et al., 2008) are both associated with higher PWB. Furthermore, those with higher education report higher wellbeing compared to the lower educated (Navarro‐Carrillo et al., 2020) and also use LTC differently: they use less informal and formal care, but more privately paid care (Abbing et al., 2021). Given that recent cohorts of 75–85‐year‐olds may suffer less from severe health impairments (Verropoulou & Tsimbos, 2017) and are higher educated than their peers in earlier cohorts (Abbing et al., 2021), we control for these variables in our analyses.

## 2 METHOD

### 2.1 Participants

For this study, data from the Longitudinal Aging Study Amsterdam (LASA) was used, an ongoing study of older adults in the Netherlands that investigates physical, cognitive, emotional and social functioning (see Hoogendijk et al., 2016 for a detailed overview of the design and recruitment procedure). Three samples of participants aged 55–84 participated in this study: sample 1 (baseline *n* = 3107) started in 1992, sample 2 was included in 2002 (baseline *n* = 1,002, 55–65‐year‐olds) in 2002 and sample 3 in 2012 (baseline *n* = 1,023, 55–65‐year‐olds). For all samples, additional measurement waves were conducted every three years. Participants were recruited in three culturally distinct regions in the west, north‐east and south of the Netherlands to reflect the national distribution of urbanisation and religiosity. Those that agreed to be interviewed were visited at home by professional interviewers who conducted regular interviews and clinical measurements that took about 2 hr to complete. The sample used for this study included participants from three cohorts in 1998, 2008 and 2018 that were living at home independently and were between 75 and 85 years old, an age group where LTC use was sufficiently prevalent. With this selection, no participant provides more than one observation, allowing independent cross‐sectional comparisons between cohorts. The total number of observations in this study was 1,456, of which 582 were included in 1998, 459 in 2005 and 415 in 2018.

### 2.2 Measures

#### 2.2.1 Outcome

Psychological wellbeing was indicated by measuring depressive symptoms, using the Center for Epidemiologic Studies Depression scale (Radloff, 1977). The scale consists of 20 statements about emotional states during the past week that participants could agree or disagree with. There were four possible answers, ranging from ‘rarely or never’ (0 Points) to ‘mostly or always’ (3 points). An example of a statement is ‘Last week I felt that everything I did costed me strength’. The score was created by adding up all points to create a scale from 0 to 60, where a higher score indicated more depressive symptoms.

#### 2.2.2 Independent variables

Care use was measured by asking participants if they receive personal and/or household care (two separate questions) and if so, from which source. They could indicate sources from 11 predefined categories. The options ‘district nurse’, elderly/home/alpha help and personal home/hospital care for either personal or domestic care indicated the use of formal care. Non‐spousal informal care was indicated by help from a child, friends, neighbours, other household members and/or other family members outside of the household. The use of privately paid care could be indicated as a separate category. Receiving care from a spouse was also an option, but we decided to use partner status instead, as having a partner is assumed to be more important for PWB than receiving care from the spouse. This information was combined to create five groups that reflect different caregiving situations: Those that have no partner and receive no care (1), those that have a partner and receive possibly care from any other source (2), those that have no partner and receive formal care with or without other informal care (3), those that have no partner and receive non‐spousal informal care only, without formal care (4), and those that have no partner and receive privately paid care, but no formal or informal care (5). These exclusive categories were chosen to clearly distinguish the groups in their impact on PWB, including potential beneficial effects of having a partner.

Care sufficiency was measured for all participants, independent of whether they receive LTC or from which source they receive it. Participants were asked to indicate whether they think that the care they receive overall is sufficient and could choose between the options ‘Yes’, ‘It is acceptable’ and ‘No’. Only the first answer indicated sufficient care provision, resulting in a binary variable.

#### 2.2.3 Control variables

Physical functioning was measured by six questions about the difficulty of daily activities based on Katz et al. (1963): Walking upstairs and downstairs, using public transport, cutting toenails, dressing and undressing, sitting down and standing up and walking outside for 5 min. Responses that indicated the difficulty of each task were measured on a 5‐point scale: (1) No, I cannot [perform this task] (2) Only with help, (3) Yes, with much difficulty, (4) Yes, with some difficulty, (5) Yes, without difficulty. The physical functioning scale was created by adding the item scores to create a scale from 6 (poor) to 30 (good functioning). Cognitive functioning was measured using the Mini‐Mental State Examination (Folstein et al., 1975). It consists of 23 items from seven categories. Each item had a maximum score of either 1, 3 or 5, resulting in a scale from 0 (poor) to 30 (good functioning). Education was measured by asking participants to indicate the highest level of education from a list of nine options which were then recoded to three education levels: Low (elementary school or no education), medium (lower vocational, intermediate education or intermediate vocational education) and high (secondary school, higher vocational education, college, or university). Gender (1 = female) and age (in years) were also included.

### 2.3 Analysis

First, descriptive statistics were included to provide an overview of sample characteristics in all three observations. Overall differences between the five care groups were analysed using ANOVAs and chi‐square tests. Post‐hoc tests that compare each group to all other groups combined were also included. Subsequently, the effects of types of LTC use (hypothesis 1) and perceived care sufficiency (hypothesis 2) on PWB were analysed with stepwise linear regression analysis for each observation (1998, 2008 and 2018) separately. Age and gender were included as controls in every model. In the first step, the LTC use variable was included as a predictor using simple contrasts, with the first group (no partner and no LTC use) as the reference category. In the second step, care sufficiency was included in the model and in the final step, education, physical and cognitive health were included. Hypotheses 1 and 2 were only tested with the final model. The stepwise approach aims at providing better insight into the explanatory effect of perceived care sufficiency and other predictors of wellbeing.

To test hypothesis 3, the full regression model was compared between the three cohorts using multigroup analysis using local estimation. The package lavaan, version 0.0.6‐6 for the statistical environment R was used (Rosseel, 2011). The model comparison was made for each independent variable between the free model and a constrained model in which that predictor was constrained. Significant differences between the effects of LTC use and care sufficiency on depressive symptoms (hypothesis 3) indicated that their effects on wellbeing have changed over time.

## 3 FINDINGS

### 3.1 Differences between the three periods

Differences in LTC use between observations were observed for all sources of LTC (see Table 1). For formal care, it differed significantly between all three cohorts, with the percentage of users decreasing over time. The percentage of privately paid care users was lower in 2008 compared to 1998, *χ* ^2^ = 8.610, *p* < .01, but did not significantly differ in 2018 compared to other cohorts. The percentage of non‐spousal informal care users was significantly lower in 2018 compared to 1998, *χ* ^2^ = 6.702, *p* < .01. Finally, the percentage that has a partner was higher in 2018 compared to 1998, *χ* ^2^ = 9.869, *p* < .01 and 2008, *χ* ^2^ = 5.654, *p* < .05. Care sufficiency was significantly higher for participants in 2018 compared to 1998, *χ* ^2^ = 5.354, *p* < .05. The percentage of higher educated differed significantly between all three cohorts, increasing over time. Physical and cognitive functioning have also improved from 1998, *t*(963.44) = −3.132, *p* < .01, and 2008, *t*(870.94) = −2.399, *p* < .05, to 2018. Depressive symptoms were on average lower in 2008, *t*(992.39) = 2.621, *p* < .01 and 2018, *t*(944.20) = 2.573, *p* < .05, compared to 1998. Overall, these differences show that the 75–85‐cohorts became healthier and higher educated, used less formal care, more often had a partner and reported higher care sufficiency and higher wellbeing over time.

**TABLE 1:** Overview of descriptive statistics for each cohort

	Diff	1998 *N* = 582	2008 *N* = 459	2018 *N* = 415
Age at interview (75–85)	c	80.3	79.9	79.6
Gender (% female)		54.8	58.8	53.5
No partner, no long‐term care use (%)		12.9	12.2	13.5
Partner (% yes)	bc	52.4	54.5	62.4
No partner, Only informal care	c	9.8	7.2	5.3
No p. Formal care or care‐mix	abc	12.7	19.4	8.2
No p. Only Privately Paid care	a	12.2	6.8	10.6
Education % high	abc	25.3	33.6	47.0
Physical functioning (6–30)	ac	25.6	25.8	26.5
Cognitive functioning (0–30)	abc	26.3	27.0	27.7
Care sufficiency (% yes)	c	79.9	84.5	87.0
Depressive symptoms (0–60)	ac	9.4	8.2	8.2

Note

a = difference 1998 and 2008 significant (*p* < .05), b = difference 2008 and 2018 significant (*p* < .05), c = difference 1998 and 2018 significant (*p* < .05)

Tables 2 and 3 provide comparisons between the average scores of each of the five groups in all three periods separately. The groups differed significantly in all measured characteristics (depressive symptoms, care sufficiency, education, physical and cognitive health, and gender distribution). The formal care users differed most from other groups due to their lower physical functioning, *t*(1453) = −12.427, *p* < .001, their lower perceived care sufficiency, *χ* ^2^ = 16.937, *p* < .01, and higher depressive symptoms, *t*(1442) = 8.439, *p* < .001. Private care users primarily differed from other groups in that they had better cognitive functioning, *t*(202.59) = 3.430, *p* < .001 and more of them were highly educated, *χ* ^2^ = 5.097, *p* < .05. In contrast, informal care users had the lowest education compared with other groups, *χ* ^2^ = 17.492, *p* < .001. In the formal, informal and privately paid care groups, a majority of participants were female, but those with a partner were primarily male, *χ* ^2^ = 245.332, *p* < .001. Those with no partner/care had the best physical functioning, *t*(405.42) = 8.710, *p* < .001. Compared with earlier cohorts, formal care users in 2018 had higher levels of depressive symptoms (15.3) and a lower prevalence of care sufficiency (64.7), which are the highest, resp. lowest values observed across all groups. In contrast, those with no partner/care or those with a partner both improved in terms of depressive symptoms and care sufficiency.

**TABLE 2:** Overview of descriptive statistics of long‐term care recipients versus non‐recipients

	1998					2008					2018				
	No Partner No Care	Partner	Formal Mix	Infor. care	Private	No Partner No Care	Partner	Formal Mix	Infor. care	Private care	No Partner No care	Partner	Formal Mix	Infor. care	Private care
	*N* = 75	*N* = 305	*N* = 74	*N* = 57	*N* = 71	*N* = 56	*N* = 250	*N* = 89	*N* = 33	*N* = 31	*N* = 56	*N* = 259	*N* = 34	*N* = 22	*N* = 44
Age (75–85)	80.0	79.7	81.0	81.1	81.3	79.2	79.4	81.5	80.4	80.5	79.2	79.2	81.1	81.0	80.0
Gender (% female)	88.0	33.1	73.0	70.2	81.7	75.0	40.8	80.9	81.8	87.1	82.1	39.8	82.4	72.7	65.9
Education (% high)	17.3	28.9	21.6	7.0	36.6	21.4	40.8	23.6	21.2	38.7	33.9	52.5	26.5	31.8	54.5
Physical functioning (6–30)	27.5	26.1	22.8	24.2	25.5	27.9	26.7	22.0	25.4	26.6	28.0	27.0	21.1	24.8	27.4
Cognitive functioning (0–30)	26.8	26.4	25.7	25.3	27.0	26.9	27.0	26.7	26.8	28.0	27.9	27.8	26.4	27.2	28.2
Care sufficiency (% yes)	80.3	84.4	71.6	84.2	80.3	84.9	85.5	80.9	93.9	90.3	89.3	90.7	64.7	81.8	84.1
Depr. symptoms (0–60)	8.8	8.22	12.05	11.3	10.6	8.1	6.8	12.0	8.6	8.5	8.3	6.9	15.3	11.3	9.1

**TABLE 3:** Overview of descriptive statistics of long‐term care recipients versus non‐recipients

	Total				
	No Partner No Care	Partner	Formal Mix	Informal care	Private care
	*N* = 187	*N* = 814	*N* = 197	*N* = 112	*N* = 146
Age at interview (75–85)	79.5	79.5	81.3	80.8	80.8
Gender (% female)	82.4	37.6	78.2	74.1	78.1
Education (% high)	23.5	40.0	23.4	16.1	42.5
Physical functioning (6–30)	27.8	26.5	22.2	24.6	26.3
Cognitive functioning (0–30)	27.2	27.1	26.3	26.1	27.6
Care sufficiency (% yes)	84.6	86.7	74.6	86.6	83.6
Depressive symptoms (0–60)	8.4	7.4	12.6	10.5	9.7

### 3.2 Main analyses

The first step of the linear regression analysis (see Table 4, Model 1) compares the effects of the five groups with no care/no partner as the reference category. Informal care use was associated with more depressive symptoms only in 1998, *b* = 3.275, *p* < .05, while formal care use was consistently associated with more depressive symptoms across cohorts (1998: *b* = 4,074, *p* < .05, 2008: *b* = 3.632, *p* < .01, 2018: *b* = 6.654, *p* < .001). Those with a partner and those who used privately paid care did not differ significantly from those with no care/no partner in terms of depressive symptoms. This pattern did not change when perceived care sufficiency was taken into account (Model 2), which suggests that these effects cannot be explained by lower care sufficiency among formal and informal care users.

**TABLE 4:** (A) The effects of long‐term care use and perceived care sufficiency on depressive symptoms

	Depressive symptoms		
	1998	2008	2018
	B	B	B
Model 1			
Age (years)	0.172	0.168	0.135
Sex (1 = female)	2.590***	1.311*	1.360*
Partner (1 = yes)	1.467	−0.692	−0.816
Only Inf. Care (1 = yes)	3.275*	0.479	2.944
Formal care (1 = yes)	4.074*	3.632**	6.654***
Only private Care (1 = yes)	2.269	0.304	0.910
Model 2			
Age (years)	0.070	0.126	0.095
Sex (1 = female)	1.779**	0.935	1.388*
Partner (1 = yes)	1.289	−0.778	−0.745
Only Inf. Care (1 = yes)	3.532**	0.977	2.678
Formal (1 = yes)	3.468**	3.598**	5.822***
Only private Care (1 = yes)	2.440*	0.659	0.740
Perceived sufficiency (1 = yes)	−6.727***	−4.613***	−4.039***
Model 3			
Age (years)	−0.038	−0.005	−0.024
Sex (1 = female)	0.812	0.097	1.337*
Partner (1 = yes)	0.188	−1.490	−1.287
Only Inf. Care (1 = yes)	1.881	−0.100	1.433
Formal (1 = yes)	1.489	1.332	3.190*
Only private Care (1 = yes)	1.819	0.492	0.682
Perceived sufficiency (1 = yes)	−6.049***	−3.196***	−2.786**
Physical functioning (6–30)	−0.448***	−0.464***	−0.396***
Cognitive functioning (0–30)	−0.028	−0.111	−0.446**
Education (0–9)	−0.314	−0.380	0.364

Note

*p < .05, **p < .01, ***p < .001.

However, when taking health and education into account (Model 3), there were no significant effects of LTC use on depressive symptoms, except that formal care increased depressive symptoms in 2018, *b* = 3.190, *p* < .05. This suggests that other effects of formal and informal care found in Model 1 and 2 can be explained by the worse physical and/or cognitive functioning or lower education in these groups. Hypothesis 1 stated that formal care use had the most negative effect on PWB, followed by informal, then by privately paid care. This hypothesis could therefore be confirmed only for the use of formal care in 2018. In 1998 and 2008, LTC use did not impact PWB when health and education are taken into account.

Care sufficiency had a significant negative effect on depressive symptoms in all observations (1998: *b* = −6.049, *p* < .001, 2008: *b* = −3.196, *p* < .001, 2018: *b* = −2.786, *p* < .01). Thus, hypothesis 2, which stated that sufficiency was associated with better wellbeing in every observation, was supported by the findings in our study.

Hypothesis 3 (differences between cohorts) was tested using multigroup analyses that compared Model 3 in 1998, 2008 and 2018. The analysis showed that the overall model differed significantly between the three observations, *χ* ^2^ = 35.365, *p* =.035. Analysing individual predictors revealed that the effect of care sufficiency on depressive symptoms differed between observations, *χ* ^2^ = 10.746, *p* < .01; the effect was more negative in 1998 compared to 2008 and 2018. Thus, those that perceived their LTC provision as sufficient displayed more positive PWB in 1998 compared to the later observations. Other independent variables did not significantly differ between cohorts, indicating a comparable effect of other sources of LTC over time.

### 3.3 DISCUSSION

The present paper investigated the impact of LTC use and the perceived sufficiency of LTC provision on the psychological wellbeing of older adults in the Netherlands in three points in time (1998, 2008, and 2018). The findings indicate that receiving formal care was associated with worse wellbeing only in 2018. In addition, having a partner and receiving either informal or privately paid care without formal care were unrelated to wellbeing in all three periods. Perceiving care provision as sufficient was consistently associated with better wellbeing, although this effect was stronger in 1998 than in the following periods.

Our hypotheses about the impact of LTC use on PWB were not supported by the findings. Unexpectedly, in 1998 and 2008, receiving LTC from any source had no impact on PWB when health and education were taken into account. Our assumption that LTC use lowers PWB due to being dependent on others may therefore not be applicable, and hint more at the distinct profile of those that received these types of care (mainly worse health). While we found that formal care recipients had significantly lower PWB in 2018 even after controlling for health and education, the multigroup comparison did not confirm that the association between formal care use and PWB differed significantly in 2018 compared to 2008 and 1998. Thus, despite the evidence that formal care recipients are in a more disadvantaged situation after the reorganisation of the LTC system in 2015, the association with PWB is empirically comparable to previous cohorts.

This finding suggests that the policy measures aiming to reduce the negative consequences of cost‐containment measures showed little effect. The enablement of client choice and increased client responsibility, for example, seem not to have changed the association between LTC use and wellbeing. It is possible, however, that there are potential negative impacts of the more recent reform that cannot yet be identified. The majority of the participants in our study might have already acquired their current LTC arrangement before the 2015 reform and might not be affected by it. Thus, it remains important to observe a potential emerging trend towards worse wellbeing when more older adults are confronted with changes in the LTC landscape. The comparable PWB might also be a result of other unobserved changes between cohorts. The lower use of LTC suggests that older adults might, for instance, have become more capable of remaining self‐sufficient. This overall positive trend towards better PWB supports this view. LTC recipients might have also maintained their wellbeing by increasingly relying on privately paid care, which is used by more participants in 2018 compared to 2008, whereas formal care use decreased (see Table 1). This also implies that those who are unable to buy LTC might be disadvantaged in this regard.

Differences in the composition of the three samples also have to be taken into consideration. Residential care has become more limited due to reforms in particularly after 2015, and the group using this type of care was not included in this study. Thus, some particularly disadvantaged individuals that would have been admitted to nursing homes in the previous periods and therefore not eligible for this study might have been included in the 2018 sample. As descriptive statistics showed, the group of formal care recipients in 2018 is indeed smaller, but more disadvantaged in terms of health and PWB. Other unmeasured characteristics of this group might therefore also affect the results (e.g. a genetic predisposition for depressive symptoms).

In contrast to the types of LTC use, perceived care sufficiency is consistently associated with better wellbeing, underlining the importance of the subjective evaluation of care provision. Despite the negative consequences that are frequently associated with LTC use, receiving sufficient support to complete daily activities and manage health‐related daily hassles plays an important role in maintaining one's wellbeing. However, this effect also weakened over time. This points to a trend towards less relevance of care sufficiency for one's PWB over time, perhaps due to an overall smaller role of the care system in the lives of older adults. Older adults in the recent cohort might be better able to cope with insufficient care provision as their expectations of the care system are lower and/or possibilities to remain self‐sufficient more widespread. Thus, while we can conclude that perceived care sufficiency is consistently essential to maintain wellbeing, a further withdrawal of the welfare state in the future might reduce this importance.

As a methodological limitation, it should be noted that for some participants, there might be reversed causality when they acquire LTC as a result of problematic psychological wellbeing. While mental health (in contrast with cognitive functioning) is not considered an eligibility criterium for publicly provided LTC in the Netherlands, participants with psychological problems might be more likely to seek help, especially from informal care sources. In addition, the operationalisation of care sufficiency in the present study can be considered limited. Having more information about the sufficiency of different aspects of LTC provision (e.g. intensity of care) would allow further disentangling factors that explain wellbeing, as well as the link between LTC use and wellbeing. These might even be factors that the participant is unaware of. For instance, someone might not consider a good client‐caregiver relationship and attentiveness important, but these factors might still contribute to his or her PWB.

Future research should therefore investigate different potential causes for care sufficiency and their impact on wellbeing. This would provide a useful foundation to design more effective policies aimed at promoting perceived care sufficiency and wellbeing. This can, for instance, help to allocate LTC resources more efficiently, for example focusing on either concentrating LTC resources on those that need it most or spread it more evenly among more LTC recipients (Rostgaard & Szebehely, 2012). Understanding how these different policy strategies impact not only care use but also perceived care sufficiency might help to choose adequate strategies to promote care sufficiency. As this study has shown, the subjective evaluation of LTC is important for maintaining PWB and should therefore be taken into consideration as much as possible to achieve the goal of providing sufficient and adequate care to everyone in need.

## STATEMENT OF ETHICAL APPROVAL

The LASA Steering Group has reviewed and approved the request for data to ensure that proposals do not violate privacy regulations and are in keeping with informed consent that is provided by all LASA participants.

## CONFLICT OF INTEREST

The authors declare no conflict of interest.

## AUTHOR CONTRIBUTIONS

Jens Abbing: Literature review, writing of the theoretical framework and discussion, data preparation and analyses.

Marjolein Broese van Groenou and Bianca Suanet: Design of the study, Feedback for revision and changes to text, advice for methodological approach, contribution of ideas.

## Data Availability

LASA data are available for research. The LASA Steering Group has adopted a policy of sharing data with interested researchers for specific research questions on aging‐related issues. To obtain data, researchers need to submit an analysis proposal that is evaluated by the LASA Steering Group. Data are available for investigation under the condition that results of analyses will be made available to the research community through scientific reports or research papers, regardless of the results of the study. More information on data requests can be found on the LASA website: www.lasa‐vu.nl.
